# The role of parental health care utilization in children’s unnecessary utilization in China: evidence from Shaanxi province

**DOI:** 10.1186/s12939-017-0544-8

**Published:** 2017-03-09

**Authors:** Yi Zhang, Zhongliang Zhou, Yafei Si

**Affiliations:** 1 Jinhe Center for Economic Research, Xi’an Jiaotong University, No. 28 Xianning West Road, Xi’an, Shaanxi 710049 China; 2 School of Public Policy and Administration, Xi’an Jiaotong University, No. 28 Xianning West Road, Xi’an, Shaanxi 710049 China

**Keywords:** Children, Parents, Health care, Utilization

## Abstract

**Background:**

China has a large population of children under 18 years of age, whose health is of great concern to the Chinese health care system. However, few studies have been conducted to analyze the factors associated with children’s unnecessary health care utilization in China. The objective of this study is to provide some empirical evidence on this issue by investigating the role of parental health care utilization in children’s unnecessary health care use.

**Methods:**

The data were obtained from the fifth Health Service Survey of Shaanxi province in 2013. We employed three dependent variables to measure children’s health care utilization: the number of children’s outpatient visits during the past 2 weeks, whether or not infusion was used if the child had any outpatient visits during the past 2 weeks, and the number of children’s inpatient visits during last year. Based on specific characteristics of these outcome variables, negative binomial models were used for the non-negative numbers of outpatient and inpatient visits, while a probit model was used for the zero-one indicator variable showing whether infusion was used during outpatient visits.

**Results:**

Based on a sample of 11,024 children, our results of multivariate analysis showed that children whose parents used outpatient care were estimated to have a larger number of outpatient visits than those whose parents did not have outpatient visits in the past 2 weeks (with a difference of 0.0393 visits). Among children having outpatient visits in the last 2 weeks, the probability of obtaining infusion was 57.01 percentage points higher for children whose parents used infusion in the past 2 weeks than the probability for those whose parents did not use infusion. The predicted number of inpatient visits was higher for children whose parents used inpatient services in the last year, compared with the group whose parents did not use (with a difference of 0.0567 visits). Moreover, we noted that the positive association between parental and children’s health care use was more prominent among younger children.

**Conclusions:**

Chinese children whose parents were high health care users were more likely to overuse health care services, holding other factors constant. Parents can play an important role in reducing children’s unnecessary outpatient visits, infusion use, and inpatient visits. The results suggest that interventions aimed at affecting patterns of parental use may be helpful in improving appropriate health care utilization for children.

## Background

According to the 2010 population census of China, children younger than 18 years of age accounted 279 million (21%) of China’s population [1]. Child health is of great concern for at least two reasons. First, children are especially vulnerable to diseases and injuries. Additionally, child health is closely related to adult health either from the perspective of epidemiology [2–4] or from the perspective of human capital accumulation [5, 6]. In recent years, a series laws and regulations, including the 1995, 2001, and 2011 Guidelines for Chinese Children’s Development [7, 8], have been implemented aiming at improving child health.

This raises the importance of promoting appropriate use of health care services among children in China. In recent years, due to the current profit-driven health care system [9–11], unnecessary use of health care services has become an increasingly severe problem in China. One case in point is the high use of antibiotics and injections [10, 12]. It was found in a study conducted in 1990 that antibiotics took about half of all prescriptions across 10 provinces in western China [13]. Based on examinations of 230,800 prescriptions written between 2007 and 2009 by 784 community health institutions in 28 cities across China, Li et al. [11] showed that in China prescriptions for antibiotics were twice more than what is recommended by the World Health Organization and rates of injection were three times higher than the estimates from other developing countries.

Moreover, there is some evidence showing that children have relatively high rates of health care use in China. Based on 2013 Analysis Report of National Health Service Survey in China [14], on average per 1000 children aged 0–4 years, the 2-week outpatient utilization rate was around 14.6% and the annual hospitalization rate was 8.6%. In contrast, the corresponding rates were 4.8% and 7.3% for adults aged 25–34 years, and 8.5% and 5.5% for adults aged 35–44 years. Besides, compared with the data from other countries, we found that Chinese children may use more health care services than their international counterparts. For example, according to the Medical Expenditure Panel Survey (MEPS), the annual rate of hospital inpatient discharges was 5% for U.S. children aged 0–4 years in 2013. Given these facts, we argue that how to reduce unnecessary use of health care for children has become a critical policy issue to be analyzed in China.

Unfortunately, few studies have been conducted to identify the factors associated with unnecessary health care utilization among Chinese children. In this paper, we aim to examine this issue by investigating the role of parental health care utilization in children’s unnecessary utilization in China. Our strategy is to compare health care utilization between the group of children whose parents were high health care users and the group whose parents were low users. In the current context of China, a positive correlation between parental and children’s health care utilization in a multivariate analysis would therefore suggest some evidence for the unnecessary use of health care services in the group of children whose parents used more health care.

We are motivated by the fact that children usually cannot make their own decisions on health care use and therefore parents, as the main caregivers, are the core decision makers on the amount and quality of health care services that children receive. Among parental attributes which may affect the health care decisions for children, parents’ own use of health care services has been shown to be a key factor for their children’s pattern of utilization.

Some early studies have presented positive correlations between parental and pediatric use of health care services. Tessler and Mechanic [15] found that children had higher use of health care services if their mothers have used more services for themselves in the past year. A study conducted by Wolfe [16] focused on a sample of children aged 1 to 11 years in New York and showed that parental use of health care services was a significant predictor for the volume of doctor visits by children. In more recent studies, such as Newacheck and Halfon [17] and Hanson [18], parental use of health care services was considered as a proxy to represent parental health beliefs and experience within the health care system. These, together with many other studies [19–26], suggested that parental health-seeking behavior and their attitudes towards the health care system would determine how their children use health care services, with higher parental use associated with higher children use. In particular, Newacheck [26] pointed out that the probability of being high users of physician services was nearly four times higher for U.S. children whose mother had high physician use than for those children whose mother had low physician use.

Parental use of health care services may play an especially important role in influencing children’s health care use in China. Different from the “individualist worldview” in the western countries, China has a “family-centered” culture. This gives Chinese parents supreme authority over their children: they are to decide children’s religion and education, manage children’s time, and make medical decisions for children [27]. There has been empirical evidence showing that Chinese parents made more decisions for children compared with American parents [28]. With respect to medical decision-making, Chinese parents not only decide whether their children would visit any health care institution, but also make the final decision whether their children would take any specific medical treatment given doctor’s suggestions. In this case, parental health beliefs and experiences are important to determine children’s health care utilization.

Previous studies also showed that income may be positively related to the use of health care services as it reflects the resources available to the family for the use of services. Gao et al. [29] found that low-income people in urban China experienced financial difficulties in seeking outpatient and inpatient care. Focusing on rural China, Wang et al. [30] and Zhou et al. [31] showed that both outpatient and inpatient visits increased in response to income growth.

Another important resource factor for health care utilization is insurance. In China, urban children are mainly covered by the Urban Resident Basic Medical Insurance Scheme (URBMI) established in 2007 and rural children are mainly covered by the New Cooperative Medical Scheme (NCMS) established in 2003. Both schemes are subsidized voluntary health insurance programs. By the end of 2013, around 2.9 billion and 8 billion people (including children) were covered by URBMI and NCMS across the country [14, 32]. The actual reimbursement rates for urban and rural residents in these two schemes were estimated to be 53.6% and 50.1% in 2013, respectively [14]. There has been evidence that these schemes have increased outpatient and inpatient utilization among the participants [33, 34]. However, as both schemes provide partial insurance, health care utilization is still dependent on income in China. Previous studies have shown that out-of pocket expense per outpatient visit has not been reduced under these programs [33, 35].

This paper contributes to the existing literature in two ways. First, to our best knowledge, this is the first study to systematically examine the potential factors of children’s unnecessary health care utilization in China. We study this issue by investigating whether parental health care utilization was associated with children’s unnecessary health care utilization. As parental influence may vary as a result of the child’s stage of development, we further examine the association between parental and children’s health care use for different age groups of children. Considering the potential problem of unnecessary health care use among Chinese children and the high involvement of Chinese parents in their children’s medical decision-making, we argue that our study has important policy implications in improving the appropriate level of children’s health care utilization in the current context of China.

Second, we analyze the parents-children relationship across several health services, including outpatient visits, infusion usage, and inpatient stays. As pointed out by Minkovitz et al. [22], extending the focus of attention from physician visits, which was the main research subject of most prior studies, to a larger set of medical services may help us obtain a more complete picture of the issue under investigation. In particular, considering the rapidly growing rates of antibiotic resistance in China [36, 37] and the risk of blood-borne virus transmission [38], we claim that analyzing infusion usage has special policy relevance at the current stage.

## Methods

### Data source

The data was obtained from the fifth National Health Service Survey (NHSS) in Shaanxi province in 2013. The NHSS was organized and conducted by the Centre for Health Statistics and Information of the Chinese Ministry of Health and has been conducted every five years since 1993. The main purpose of this survey is to collect information on health care need, utilization, quality, and expenditure, to evaluate the performance of health reform and development of the previous five years, and to provide proofs for China’s health care planning, targets, and major action plans. The survey data has been used in many previous studies [30, 35, 39–41].

Representative samples were obtained through a four-stage stratified random sampling procedure before any individual interview was conducted [14]. In the first stage, 32 out of total 107 counties or districts of Shaanxi Province were randomly selected into the sample. In the second stage, 5 townships or streets were randomly chosen from each of the selected counties or districts. In the third stage, 2 villages or residents’ communities in each selected township were randomly selected. In the last stage, 60 households were randomly selected in each sampled village/community. Finally, 20,700 households were identified.

The NHSS questionnaire mainly includes household demographics, socioeconomic status, self-reported health status, and health care utilization. Qualified investigators were employed in order to collect household data. They visited sample households and interviewed each household member face-to-face one by one. Data were collected from 57,529 respondents. Residents were required to answer questions themselves. Information on children was collected through their parents. The adult in-person response rate was 82.1%.

Quality control was implemented through the survey. The investigators were mainly local medical workers and investigation instructors were mainly doctors from township hospitals or health institutions and above. Investigation instructors revisited 5% of the sample households and asked 14 key questions again in order to check the accuracy of data recorded by investigators. The compliance rate after countercheck was over 97.7%. Various survey data tests, including the Myer’s index, the goodness-of-fit test, DELTA dissimilarity coefficient and GINI concentration ratio, were applied to check data quality and consistency. Results showed: the survey had no age preference; the sampled age distribution was not significantly different from the overall distribution; the size of surveyed households was consistent with that of the population.

For the purpose of this study, we identified 11,024 children under 18 years of age. To analyze the relation between parental and children’s health care use, each of these children was paired with one parent. Given the design of the survey questionnaire, we could only obtain the parental information from the parent who was the head-of-household. Among the identified parents, 72% were male (fathers) and 28% were female (mothers). Though we were only able to obtain information on one parent from the survey, this limitation was not serious since it is the head-of-household, usually the father, who has final authority in a Chinese family and participates actively in medical decision-making [27].

### Dependent variables

#### Children’s health care utilization

In the survey, parents were asked to answer separate questions regarding their children’s outpatient and inpatient visits during certain periods preceding the interview. We selected the following three variables on a set of children’s use of health care services (a detailed description of the dependent variables was provided in Table 1):

**Table 1:** Description of dependent variables

Dependent variable	Explanation
Outpatient visit	An outpatient visit was defined in the survey as a patient visit to hospital outpatient facilities in a day and included pharmaceuticals, physician visits, and outpatient surgery (excluding routine well-child visits such as general checkups and immunizations). The leading causes of outpatient visits among the surveyed children in the past 2 weeks were: common cold, upper respiratory tract infection, and influenza.
Infusion	The infusion variable was identified as being conditional on having an outpatient visit. The leading causes of obtaining infusion during outpatient visits in the past 2 weeks were: upper respiratory tract infection, common cold, and influenza.
Inpatient visit	An inpatient visit was defined as a patient visit which resulted in overnight hospitalization. The leading causes of inpatient visits among the surveyed children in the last year included: upper respiratory tract infection, pneumonia, and acute nasopharyngitis.

Note: Following previous studies on the utilization of health care services, self-reported responses were used in this study. The period of recall was 2 weeks for outpatient utilization and one year for inpatient utilization in the NHSS. Although one disadvantage of using self-reported data is that they are subject to reporting bias, using a relatively short recall period would help reduce the likelihood of recall error [64]

1. Outpatient visits: Number of outpatient visits in the past 2 weeks (continuous; including zero for never).
2. Infusion: Obtaining infusion during outpatient visits in the past 2 weeks (Yes = 1, No = 0).
3. Inpatient visits: Number of inpatient visits in the last year (continuous; including zero for never).

### Independent variables

The independent variables along with their explanations were illustrated in Table 2. Variables on parental health care utilization reflected parental experience within the health care system and were used to proxy parental health-seeking behavior and their attitudes towards the health care system. We hypothesized that children would be more likely to be high users of health care services when parental health care use was high. In order to capture the independent effect of parental health care use on children’s unnecessary use, we also included a set of variables that might be related to parental and children’s health care use. Applying the Aday and Anderson framework [42], we included predisposing factors such as age, gender, and family structure [19, 43–46], need factors related to children’s need for care [19–21, 44], as well as enabling factors like family income and insurance coverage [47, 48].

**Table 2:** Description of independent variables

Independent variable	Explanation	Note
Parental health care utilization	1. Parental outpatient visit: The paired parent used outpatient services in the past 2 weeks (Yes = 1, No = 0) 2. Parental infusion use: The paired parent used infusion during outpatient visits in the past 2 weeks (Yes = 1, No = 0) 3. Parental inpatient visit: The paired parent used inpatient services in the last year (Yes = 1, No = 0)	Binary instead of count variables were employed, since only 1.71% of the surveyed parents used outpatient services more than once in the past 2 weeks and 0.46% used inpatient services more than once in the last year.
Parental socioeconomic level	Per capita household expenditure last year (five equal quantiles for the poorest, poor, average, rich, and the richest)	It has shown to be a better proxy for resources available than self-reported income which is more likely to be misreported [65, 66].
Children’s health insurance	A child was covered by any insurance scheme (Yes = 1, No = 0)	Among the surveyed children, 91.6% of the urban children were covered by URBMI and 96.9%% of the rural children were covered by NCMS. Since whether a child was under URBMI or NCMS depended on his/her residential area controlled later, we used an indicator variable for insurance status instead of specifying different insurance schemes.
Children’s need for care	1. Days of illness if a child was reported to have any illnesses or injuries in the past 2 weeks 2. Children’s height and weight (self-reported)	We used the days of illness to indicate the severity of the illness and therefore the need for health care services. As the recall period for the days of illness was 2 weeks, this variable was not able to be taken into account in the analysis of the use of inpatient services in the last year.
Parental need for care	Days of illness if the parent was reported to have any illnesses or injuries in the past 2 weeks	Due to time inconsistency, this variable was not used in the inpatient model.
Children’s age	Grouped into 0–2, 3–5, 6–8, 9–11, 12–14, 15–17 years	
Children’s gender	Male or female	
Children’s ethnicity	Han or others	
Parental age	Parental age in the survey year	
Residential areas	Urban or rural	
Living region	Shanbei (north), Guanzhong (central), and Shannan (south)	
Family size	The number of members in a family	

Note: Though parental education has been shown to be associated with children’s health care utilization in some previous studies [17, 22], we did not include this variable as it was highly correlated with parental income in our sample

### Estimation methods

In this study, we examined each outcome of utilization separately. According to the Anderson health behavior model for the study of health services utilization [42, 49], it is important to specify the relevant dimension of utilization, as each may differ in type, purpose as well as the time interval involved and hence reflect different aspects of the health care seeking process. We employed two different econometric models for estimation based on the specific features of these dependent variables. For outpatient visits and inpatient visits which were measured by non-negative numbers, negative binomial regressions were adopted for modeling count outcome variables. Compared with the ordinary least squares (OLS) regression models, negative binomial models are better fitted to our utilization data as they allow for zero outcomes and can better deal with heteroscedasticity [50]. Compared with poisson regression models, negative binomial models fit the data better when there is overdispersion. For infusion use which was measured by a zero-one indicator variable, we employed a probit model to estimate the probability that a child with particular characteristics was treated with infusion during outpatient visits. The probit model is well known as a better candidate to model binary outcome variables than a linear probability model, as the homoskedasticity and normality assumptions of OLS regression are violated in the latter method [51].

As both negative binomial and probit models are nonlinear models, we take a linear approximation of the model functions. The marginal effects were calculated from predictions of a model at the means of the independent variables [52]. The regression model was specified as below:

1 $$ {Y}_i=\alpha +{\displaystyle {\sum}_j{\beta}_j{X}_{i j}+{\displaystyle {\sum}_k{\gamma}_k{Z}_{i k}+{\varepsilon}_i}} $$

In the model above, *Y*_*i*_ represents for dependent variables of children’s health care utilization (i.e., outpatient visits, infusion use during outpatient visits, or inpatient visits); *X*_*ij*_ capture the main explanatory variables including parental care utilization, parental socioeconomic status, children’s health insurance, children’s need for care, and parental need for care; *Z*_*ik*_ are all the other control variables; *ε*_*i*_ denotes the error term. In particular, we were interested in the impact of parental utilization on children’s health care use. Following the theoretical framework in Aday and Anderson [42] and the empirical work of Minkovitz et al. [22], the three indicator variables on parental utilization separately entered into three regression models with the number of children’s outpatient visits, whether or not infusion was used during outpatient visits for children, and the number of children’s inpatient visits as dependent variables, respectively. Considering that the importance of the parental influence might vary according to the development stage of the child, the full sample was further split into three age groups, which represent pre-school (0–5), primary education (6–11), and secondary education age (12–17), respectively. The coefficients *β*_*j*_ and *γ*_*k*_ were partical effects (*dY/dX*_*j*_ and *dY/dZ*_*k*_) of the independent variables on the predicted count or probability of events. The analyses were performed using STATA software (version 13.1; Stata Corporation, College Station, TX).

## Results

### Descriptive analysis

Summary statistics for both dependent and independent variables were reported in Table 3. This study included 11,024 children under 18 years. Among them, 6.37% had at least one outpatient visit in the last 2 weeks and 5.6% experienced at least one hospital stay in the last year. The sample for the analysis of infusion use included 702 children who used outpatient services at least once in the last 2 weeks. Among them, 38.46% received the infusion treatment. In the last 2 weeks, the surveyed children had on average 0.4417 days of illness. With respect to parental health care utilization in the sample (10,696 parents included), 5.96% had at least one outpatient visit in the last 2 weeks, 36.73% obtained infusion during outpatient visits, and 6.95% had at least one inpatient visit in the last year. The average age of parents was around 34 years. The average number of days of illness was 0.9204 days for parents. The average level of per capita household expenditure in the sample ranged from 2628 Yuan (poorest) to 16207 Yuan (richest) over the last year. The majority of the surveyed children were covered by insurance (94.91%) and of Han ethnicity (98.27%). There were relatively more male (53.3%) and rural (62.13%) children in the sample.

**Table 3:** Descriptive statistics for variables

Variable	Number	Mean (S.D.) or %
Number of children’s outpatient visit	11,024	0.0907 (0.4173)
0	10,322	93.63
1	526	4.77
2	101	0.92
3 and more	75	0.68
Children’s infusion use (yes or no)		
No	432	61.54
Yes	270	38.46
Number of children’s inpatient visit	11,024	0.0788 (0.3844)
0	10,407	94.4
1	468	4.25
2	82	0.74
3 and more	67	0.61
Parental outpatient visit		
No^a^	10,059	94.04
Yes	637	5.96
Parental infusion use		
No^a^	403	63.27
Yes	234	36.73
Parental inpatient visit		
No^a^	9,385	93.05
Yes	701	6.95
Parental days of illness	10696	0.9204 (3.0819)
Parental age	10696	34.15 (12.75)
Per capita household expense		
Poorest^a^	2,208	2628 (737)
Poor	2,206	4503 (470)
Average	2,230	6291 (570)
Rich	2,186	8752 (938)
Richest	2,205	16207 (6686)
Children’s insurance		
No^a^	560	5.09
Yes	10,448	94.91
Children’s days of illness	11035	0.4417 (1.8273)
Children’s height (cm)	10986	125.12 (34.24)
Children’s weight (kg)	10985	32.49 (17.20)
Children’s age		
0-2^a^	1,992	18.07
3–5	1,941	17.61
6–8	1,650	14.97
9–11	1,527	13.85
12–14	1,700	15.42
15–18	2,214	20.08
Gender		
Female^a^	5,153	46.7
Male	5,882	53.3
Ethnicity		
Others^a^	191	1.73
Han	10,842	98.27
Residential area		
Rural^a^	6,856	62.13
Urban	4,179	37.87
Region		
Shannan^a^	4,074	36.92
Guanzhong	5,100	46.22
Shanbei	1,861	16.86
Family size	11035	4.01 (1.15)

Note: “Number” represents the number of observations of a variable

^a^Reference levels in the regressions; Outpatient visits and infusion in the past 2 weeks; Inpatient visits in the last year

**Fig. 1:**
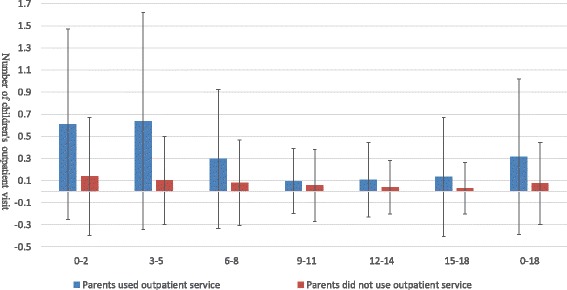
Average number of children’s outpatient visit when parents used/did not use outpatient services in the past 2 weeks

**Fig. 2:**
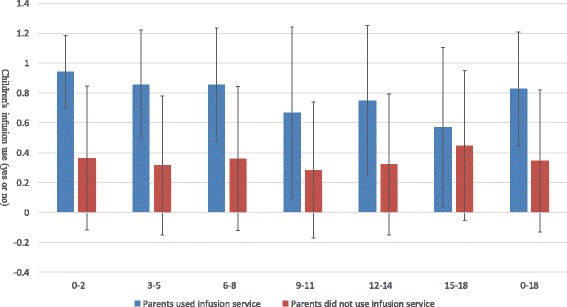
Average probability of children’s infusion use during outpatient visits when parents obtained/did not obtain infusion during outpatient visits

**Fig. 3:**
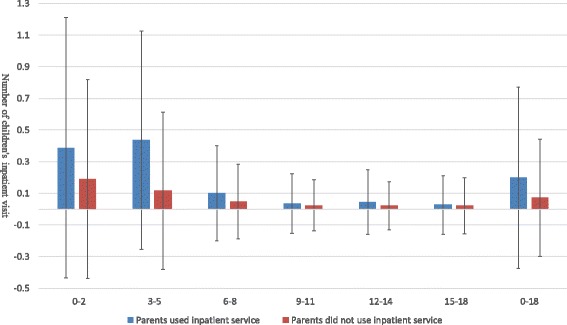
Average number of children’s inpatient visit when parents used/did not use inpatient services in the last year

Figures 1, 2 and 3 illustrated the association between parental and children’s medical services utilization. In the past 2 weeks, children whose parents had at least one outpatient visit had more outpatient visits than those whose parents never used outpatient services. During outpatient visits, children whose parents obtained infusion were more likely to use infusion than those whose parents did not obtain infusion. In the last year, children whose parents had at least one inpatient visit had more inpatient visits than those whose parents had no hospital stays. Similar results could be found for different age groups of children. These findings provided some evidence for a positive association between parental and children’s health services utilization.

### Regression results

Tables 4, 5 and 6 reported the results of multivariate analysis. Estimated marginal effect, standard error, and 95% confidence interval were reported for each independent variable. In multivariate regressions, we incorporated all variables illustrated in Table 2 so as to better ensure the estimated association between parental and children’s health care use was independent of the effects of other confounding factors. The dependent variable was the number of children’s outpatient visits during the past 2 weeks in Table 4, whether or not infusion was used during outpatient visits in Table 5, and the number of children’s inpatient visits during the last year in Table 6, respectively. Due to missing covariates, sample sizes were reduced in regressions. We checked the consistency between the observations used for the analyses and those in the full sample applying the chi-square test. The test result showed that our estimation results were not affected by the missing covariates. In each table, we showed the estimation results for the full sample and those for different age groups (0–5, 6–11, and 12–17). However, we should also bear in mind that focusing on different age groups largely reduced the number of observations for each subsample, which was likely to reduce the estimation efficiency and the reliability of the results. This was especially the case when we analyzed the use of infusion, for which the full sample size was already relatively small.

**Table 4:** Negative binomial regression results on the use of outpatient services in the past 2 weeks

	Number of children’s outpatient visit in the last 2 weeks (marginal effect reported)															
	(1) Full sample				(2) Age 0–5				(3) Age 6–11				(4) Age 12–18			
	dy/dx	S.E.	95% CI		dy/dx	S.E.	95% CI		dy/dx	S.E.	95% CI		dy/dx	S.E.	95% CI	
Parents had outpatient visit	0.0393^a^	0.0106	0.0186	0.0600	0.0980^a^	0.0306	0.0381	0.1579	0.0170	0.0146	−0.0117	0.0457	0.0179^c^	0.0107	−0.0031	0.0390
Per capita household expense																
Poor	−0.0010	0.0036	−0.0080	0.0061	0.0057	0.0099	−0.0138	0.0251	0.0007	0.0061	−0.0113	0.0127	−0.0051^c^	0.0031	−0.0111	0.0008
Average	−0.0023	0.0036	−0.0094	0.0048	−0.0048	0.0091	−0.0228	0.0131	0.0003	0.0065	−0.0124	0.0129	−0.0031	0.0038	−0.0106	0.0044
Rich	−0.0016	0.0036	−0.0087	0.0055	0.0040	0.0107	−0.0170	0.0251	−0.0079	0.0053	−0.0183	0.0025	−0.0024	0.0037	−0.0096	0.0048
Richest	−0.0002	0.0039	−0.0078	0.0075	0.0111	0.0118	−0.0119	0.0342	−0.0086	0.0062	−0.0207	0.0034	0.0008	0.0044	−0.0078	0.0094
Children had insurance	0.0005	0.0040	−0.0074	0.0084	0.0000	0.0098	−0.0191	0.0192	−0.0090	0.0116	−0.0318	0.0137	0.0174^a^	0.0025	0.0125	0.0224
Children’s days of illness	0.0133^a^	0.0008	0.0118	0.0147	0.0241^a^	0.0013	0.0216	0.0267	0.0122^a^	0.0013	0.0096	0.0148	0.0045^a^	0.0005	0.0035	0.0055
Children’s height (ln)	0.0034	0.0074	−0.0110	0.0179	0.0128	0.0163	−0.0191	0.0447	−0.0156	0.0175	−0.0498	0.0187	0.0047	0.0142	−0.0231	0.0326
Children’s weight (ln)	−0.0010	0.0039	−0.0086	0.0066	−0.0053	0.0083	−0.0215	0.0109	0.0108	0.0087	−0.0062	0.0279	−0.0051	0.0072	−0.0191	0.0090
Prenatal days of illness	−0.0013^b^	0.0005	−0.0023	−0.0002	−0.0033^b^	0.0015	−0.0062	−0.0004	−0.0001	0.0008	−0.0016	0.0014	−0.0009^c^	0.0005	−0.0018	0.0000
Parental age	0.0001	0.0001	−0.0001	0.0002	0.0003	0.0002	−0.0001	0.0006	−0.0001	0.0002	−0.0004	0.0003	0.0001	0.0002	−0.0002	0.0004
Children’s age																
3-5	−0.0051^c^	0.0030	−0.0110	0.0008	−0.0126^c^	0.0073	−0.0269	0.0017								
6-8	−0.0074^c^	0.0040	−0.0152	0.0005												
9-11	−0.0161^a^	0.0039	−0.0237	−0.0085					−0.0100^b^	0.0048	−0.0194	−0.0007				
12-14	−0.0154^a^	0.0043	−0.0238	−0.0069												
15-18	−0.0239^a^	0.0048	−0.0333	−0.0145									−0.0078^b^	0.0036	−0.0148	−0.0007
Gender was male	−0.0031	0.0023	−0.0075	0.0014	−0.0095^c^	0.0056	−0.0206	0.0015	0.0025	0.0041	−0.0055	0.0106	−0.0036	0.0026	−0.0086	0.0015
Ethnicity was Han	−0.0004	0.0099	−0.0198	0.0191	0.0022	0.0275	−0.0517	0.0560	0.0023	0.0170	−0.0310	0.0357	0.0005	0.0105	−0.0200	0.0210
Urban	0.0095^a^	0.0028	0.0039	0.0150	0.0113 ^c^	0.0067	−0.0018	0.0244	0.0190^a^	0.0063	0.0067	0.0314	−0.0017	0.0029	−0.0074	0.0041
Region																
Guanzhong	−0.0006	0.0026	−0.0056	0.0044	−0.0011	0.0061	−0.0131	0.0109	−0.0015	0.0045	−0.0104	0.0073	0.0023	0.0030	−0.0036	0.0083
Shanbei	−0.0033	0.0030	−0.0092	0.0026	−0.0117	0.0079	−0.0272	0.0038	0.0012	0.0057	−0.0100	0.0124	−0.0037	0.0039	−0.0113	0.0039
Family size	0.0022^b^	0.0011	0.0001	0.0042	0.0047^c^	0.0026	−0.0004	0.0099	0.0012	0.0017	−0.0022	0.0046	0.0014	0.0012	−0.0010	0.0038
No. of obs.	10,596				3,732				3,077				3,787			

Note: ^a^Statistically significant at the 1% level, ^b^statistically significant at the 5% level, ^c^statistically significant at the 10% level; S.E. robust standard errors; marginal effects instead of the original coefficients are reported to show the marginal change of the dependent variable given a one unit change in an explanatory variable. The reference groups were: “the poorest” for “per capita household expense”; “no” for “children had insurance”; “0-2” for “children’s age”; “female” for “gender was male”; “others” for “ethnicity was Han”; “rural” for “urban”; “Shannan” for “region”

**Table 5:** Negative binomial regression results on the use of infusion during outpatient visits in the past 2 weeks

	Whether or not children used infusion during outpatient visits (marginal effect reported)															
	(1) Full sample				(2) Age 0-5				(3) Age 6-11				(4) Age 12-18			
	dy/dx	S.E.	95% CI		dy/dx	S.E.	95% CI		dy/dx	S.E.	95% CI		dy/dx	S.E.	95% CI	
Parents had outpatient visit	0.5701^a^	0.0518	0.4686	0.6716	0.6624^a^	0.0412	0.5817	0.7431	0.5447^b^	0.1527	0.2454	0.8441	0.3005	0.1874	−0.0668	0.6679
Per capita household expense																
Poor	0.1114	0.0713	−0.0284	0.2512	0.2073^b^	0.1036	0.0042	0.4104	0.1329	0.1337	−0.1291	0.3948	−0.172	0.1500	−0.4660	0.1221
Average	0.0243	0.0734	−0.1194	0.1681	0.1086	0.1137	−0.1141	0.3314	−0.1129	0.1279	−0.3635	0.1377	−0.0308	0.1764	−0.3765	0.3150
Rich	0.1145	0.0769	−0.0362	0.2652	0.1482	0.1089	−0.0651	0.3616	0.0696	0.1790	−0.2812	0.4204	0.1024	0.1816	−0.2535	0.4583
Richest	0.2171^a^	0.0768	0.0665	0.3677	0.2940^a^	0.1061	0.0860	0.5020	0.4491^a^	0.1494	0.1564	0.7418	−0.2679^a^	0.1343	−0.5312	−0.0046
Children had insurance	0.0162	0.0798	−0.1402	0.1725	0.0134	0.0898	−0.1627	0.1895	0.2024	0.1986	−0.1869	0.5916				
Children’s days of illness	0.0170^a^	0.0059	0.0054	0.0286	0.0270^a^	0.0090	0.0093	0.0447	0.0129	0.0119	−0.0104	0.0363	−0.0039	0.0126	−0.0287	0.0208
Children’s height (ln)	−0.3658^b^	0.1470	−0.6538	−0.0777	−0.3580^b^	0.1696	−0.6903	−0.0256	−0.2018	0.4052	−0.9959	0.5924	−3.1048^a^	1.0277	−5.1190	−1.0905
Children’s weight (ln)	0.1626^b^	0.0664	0.0324	0.2928	0.1033	0.0731	−0.0400	0.2465	0.2773	0.2061	−0.1267	0.6813	0.6444	0.4297	−0.1978	1.4866
Prenatal days of illness	−0.0136^b^	0.0057	−0.0247	−0.0024	−0.0252^a^	0.0083	−0.0415	−0.0089	−0.0112	0.0126	−0.0358	0.0134	−0.0036	0.0119	−0.0268	0.0197
Parental age	0.0030^a^	0.0017	−0.0003	0.0063	0.0047^b^	0.0022	0.0004	0.0089	−0.0059	0.0044	−0.0144	0.0027	0.0001	0.0071	−0.0139	0.0141
Children’s age																
3–5	−0.0299	0.0623	−0.1520	0.0923	−0.0079	0.0695	−0.1441	0.1284								
6–8	0.0261	0.0896	−0.1496	0.2018												
9–11	−0.0916	0.1032	−0.2939	0.1108					−0.1808^a^	0.0965	−0.3698	0.0083				
12–14	−0.0229	0.1298	−0.2774	0.2315												
15–18	0.0245	0.1472	−0.2639	0.3130									0.2925^b^	0.1142	0.0687	0.5162
Gender was male	0.0431	0.0402	−0.0356	0.1219	0.0601	0.0560	−0.0496	0.1698	−0.0191	0.0822	−0.1803	0.1421	0.0962	0.1042	−0.1229	0.2857
Ethnicity was Han	−0.1443	0.2482	−0.6308	0.3422	−0.0702	0.2942	−0.6468	0.5064								
Urban	0.0476	0.0455	−0.0417	0.1368	0.0418	0.0623	−0.0803	0.1639	0.0186	0.0988	−0.1751	0.2123	0.0962	0.1209	−0.1409	0.3332
Region																
Guanzhong	−0.0127	0.0470	−0.1049	0.0795	−0.0106	0.0650	−0.1380	0.1167	−0.0644	0.0999	−0.2601	0.1313	−0.1179	0.1093	−0.3321	0.0963
Shanbei	−0.0184	0.0603	−0.1366	0.0999	0.0066	0.0808	−0.1517	0.1650	−0.2344^b^	0.0960	−0.4225	−0.0463	0.0728	0.1741	−0.2685	0.4141
Family size	0.0267	0.0179	−0.0084	0.0618	0.0394	0.0256	−0.0108	0.0897	0.0853^b^	0.0357	0.0152	0.1553	−0.1122^b^	0.0497	−0.2096	−0.0148
No. of obs.	672				391				164				115			

Note: ^a^Statistically significant at the 1% level, ^b^statistically significant at the 5% level, ^a^statistically significant at the 10% level; S.E. robust standard errors; marginal effects instead of the original coefficients are reported to show the marginal change of the dependent variable given a one unit change in an explanatory variable. The reference groups were: “the poorest” for “per capita household expense”; “no” for “children had insurance”; “0–2” for “children’s age”; “female” for “gender was male”; “others” for “ethnicity was Han”; “rural” for “urban”; “Shannan” for “region”

**Table 6:** Negative binomial regression results on the use of inpatient services in the last year

	Number of children’s inpatient visit in the last year (marginal effect reported)															
	(1) Full sample				(2) Age 0–5				(3) Age 6–11				(4) Age 12–18			
	dy/dx	S.E.	95% CI		dy/dx	S.E.	95% CI		dy/dx	S.E.	95% CI		dy/dx	S.E.	95% CI	
Parents had outpatient visit	0.0567^a^	0.0104	0.0364	0.0770	0.2419^a^	0.0462	0.1514	0.3324	0.0239	0.0163	−0.0080	0.0558	0.0009	0.0056	−0.0101	0.0119
Per capita household expense																
Poor	0.0187^b^	0.0080	0.0031	0.0343	0.1042^a^	0.0353	0.0349	0.1735	−0.0058	0.0066	−0.0188	0.0072	0.0022	0.0048	−0.0073	0.0116
Average	0.0248^a^	0.0090	0.0072	0.0425	0.1355^a^	0.0436	0.0500	0.2210	−0.0036	0.0072	−0.0177	0.0105	0.0035	0.0051	−0.0065	0.0134
Rich	0.0419^a^	0.0111	0.0200	0.0637	0.1910^a^	0.0492	0.0945	0.2875	−0.0106	0.0076	−0.0255	0.0044	0.0191^b^	0.0092	0.0011	0.0372
Richest	0.0331^a^	0.0103	0.0130	0.0532	0.1533^a^	0.0464	0.0623	0.2442	−0.0082	0.0074	−0.0227	0.0063	0.0133^c^	0.0071	−0.0007	0.0273
Children had insurance	0.0292^a^	0.0049	0.0195	0.0388	0.0941^a^	0.0170	0.0609	0.1274	0.0078	0.0156	−0.0227	0.0383	0.0166^a^	0.0024	0.0118	0.0214
Children's height (ln)	0.0040	0.0157	−0.0269	0.0348	−0.0107	0.0500	−0.1087	0.0873	0.0545^b^	0.0232	0.0090	0.1000	−0.0153	0.0159	−0.0465	0.0160
Children's weight (ln)	−0.0228^a^	0.0085	−0.0395	−0.0060	−0.0310	0.0269	−0.0838	0.0217	−0.0591^a^	0.0115	−0.0816	−0.0366	−0.0011	0.0078	−0.0163	0.0142
Parental age	0.0004^b^	0.0002	0.0001	0.0007	0.0008	0.0005	−0.0002	0.0018	0.0008^a^	0.0002	0.0003	0.0012	0.0001	0.0002	−0.0002	0.0004
Children's age																
3-5	−0.0114^c^	0.0058	−0.0227	0.0000	−0.0446^b^	0.0213	−0.0863	−0.0028								
6-8	−0.0352^a^	0.0054	−0.0458	−0.0246												
9-11	−0.0467^a^	0.0053	−0.0571	−0.0362					−0.0113^c^	0.0065	−0.0241	0.0014				
12-14	−0.0480^a^	0.0066	−0.0610	−0.0351												
15-18	−0.0545^a^	0.0077	−0.0697	−0.0394									−0.0008	0.0029	−0.0065	0.0048
Gender was male	0.0116^a^	0.0041	0.0036	0.0196	0.0213	0.0153	−0.0086	0.0512	0.0130^b^	0.0055	0.0022	0.0237	0.0054^b^	0.0026	0.0003	0.0105
Ethnicity was Han	0.0217^b^	0.0104	0.0014	0.0421	0.0781^b^	0.0341	0.0113	0.1448	0.0080	0.0193	−0.0298	0.0458	−0.0029	0.0135	−0.0293	0.0235
Urban	−0.0113^a^	0.0043	−0.0197	−0.0029	−0.0342^b^	0.0162	−0.0659	−0.0024	−0.0044	0.0059	−0.0158	0.0071	−0.0030	0.0025	−0.0079	0.0019
Region																
Guanzhong	0.0194^a^	0.0049	0.0099	0.0289	0.0482^a^	0.0176	0.0136	0.0827	0.0278^a^	0.0080	0.0121	0.0435	0.0028	0.0027	−0.0024	0.0080
Shanbei	−0.0054	0.0062	−0.0176	0.0068	−0.0368^c^	0.0214	−0.0789	0.0052	0.0359^b^	0.0156	0.0053	0.0665	−0.0084^a^	0.0026	−0.0134	−0.0034
Family size	0.0068^a^	0.0020	0.0030	0.0106	0.0326^a^	0.0077	0.0176	0.0476	−0.0041^c^	0.0024	−0.0088	0.0007	0.0024^b^	0.0010	0.0005	0.0044
No. of obs.	9,998				3,546				2,918				3,534			

Note: ^a^Statistically significant at the 1% level, ^b^statistically significant at the 5% level, ^c^statistically significant at the 10% level; S.E. robust standard errors; marginal effects instead of the original coefficients are reported to show the marginal change of the dependent variable given a one unit change in an explanatory variable. The reference groups were: “the poorest” for “per capita household expense”; “no” for “children had insurance”; “0–2” for “children’s age”; “female” for “gender was male”; “others” for “ethnicity was Han”; “rural” for “urban”; “Shannan” for “region”

In Table 4, for the full sample, holding other variables constant, the predicted number of outpatient visits was on average higher for the group of children whose parents had outpatient visits during the past 2 weeks, compared to the group whose parents did not have outpatient visits (with a difference of 0.0393 visits). The estimated difference was statistically significant. We also found that the number of children’s outpatient visits was significantly positively associated with their days of illness during the past 2 weeks. For the full sample, given a 1 day increase in the number of illness days, children were estimated to have 0.0133 more outpatient visits. Children’s use of outpatient services was not significantly related to per capita household expense or the insurance status of children. Parental days of illness had a significantly negative effect on the number of children’s outpatient visits. Among all the age groups of children, the number of outpatient visits significantly decreased with children’s age. Compared with rural children, urban children visited the outpatient department more frequently (0.0095 more visits in the full sample). Children from larger families reported a larger number of outpatient visits. These differences were statistically significant.

In Table 5, a child had a higher probability of obtaining infusion during outpatient visits if his/her parent used infusion in the past 2 weeks (57.01 percentage points higher). The difference was statistically significant for children aged 0–11 years. For children aged 12–17, children’s use of infusion during outpatient visits was not significantly associated with parental use of infusion. Among young children aged 0–5 years, the probability of using infusion during outpatient visits in the past 2 weeks was significantly positively correlated with their days of illness, but significantly negatively correlated with parental days of illness. We found that elder parents were significantly more inclined to have their children receive infusion treatment. In addition, there was some mild evidence that the probability of using infusion during outpatient visits was positively correlated with per capita household expense.

In Table 6, for the full sample, children with parents who were hospitalized last year were estimated to have a larger number of inpatient visits than those whose parents used no inpatient services last year. The difference was 0.0567 visits and statistically significant. The positive association between parental and children’s use of inpatient services was only significant among children aged 0–5 years. Per capita household expenditure was significantly related to the use of inpatient services. Children with insurance coverage were found to have a statistically significant larger number of inpatient visits compared to those being uninsured. The results also showed that older children had fewer inpatient visits in the last year. The predicted number of inpatient visits was higher for boys than that for girls (boys had 0.0116 more visits in the full sample). Relative to the base group of the southern region (Shannan), children living in the central part of the province (Guanzhong) had more visits for inpatient care (0.0194 more visits in the full sample). Children living in the rural area had a larger number of inpatient visits (0.0113 more visits in the full sample) than those living in the urban area. These differences were all statistically significant. We also found that the number of inpatient visits was significantly positively correlated with parental age and the size of the family.

## Discussion

This study provided empirical evidence for the association between parental and children’s unnecessary health care utilization in China. We found that children, especially young children, used more health care services if their parents themselves used more health care services. This positive correlation was found in a set of healthcare services, including outpatient care, infusion during outpatient visits, and hospitalization, after controlling for various individual, parental, and family characteristics. The existing literature on the association between parental and children’s health care use has been mainly focused on US children. Few relevant studies could be identified for children in the developing world. Our results are consistent with previous US-based findings, though based on different sample and methodology, with respect to outpatient care [17–19, 22] and inpatient visits [22]. In addition, compared with previous studies, we reported new evidence on the association between parental and children’s health care use for different age groups of children and for the utilization of infusion.

In this study, parental use of health care services was used to represent parental health-seeking behavior and their attitudes towards the health care system. The results supported our hypothesis that parents who used more health care services tended to have their children receive more medical services as well. The association between parental and children’s health care utilization implies that programs and policies designed to change parental health-seeking behaviors may be effective in reducing unnecessary use of health care services among children. In particular, providing parents with sufficient health-related knowledge, for example on the pros and cons of using infusion, may reduce overuse of antibiotics and risks related with acupuncture and blood transfusion [10, 38], which exert adverse consequences on child health [53]. Moreover, as the positive association between parental and children’s health care use was found to be more significant among young children, policy or educational interventions targeting parents of young children could be more efficient.

Compared with uninsured children, children covered by insurance had more inpatient visits. Since both URBMI for urban children and NCMS for rural children were designed to reduce the risk of catastrophic health spending and did not cover (or partially covered) outpatient services, insurance status was not an important predictor for children’s use of outpatient care. On the one hand, insurance might help increase the likelihood of children seeking inpatient care as a proportion of the inpatient expense was reimbursed. On the other hand, previous studies have shown that medical insurance, combined with fee-for-service provision and provider bonuses, might encourage the use of more expensive health care services [54, 55]. Such insurance-induced moral hazard might be related to the overuse of health care services. However, using the current data in this study, we were not able to provide direct evidence to clarity this issue.

We found that per capita household expense was positively associated with children’s use of inpatient services in the last year, while it had no effect on children’s use of outpatient services in the past 2 weeks. The results are consistent with findings in Zhou et al. [31], which calculated the income elasticity of healthcare demand in rural China and found a faster growth of inpatient services than outpatient services in response to income growth. Per capita household expense was significantly positive even after controlling for insurance status in the inpatient model. The finding might be related to the fact that even for inpatient care, both URBMI and NCMS had high deductibles, low ceilings, and high coinsurance rates [33, 34]. The results imply that programs reducing unnecessary health care utilization among children could be more effective if targeted at expensive health care services.

Measured by the days of illness in the past 2 weeks, children’s need for care was found to be positively associated with the number of outpatient visits in the past 2 weeks and the probability of using infusion during outpatient visits. The results are consistent with previous studies using children’s general health status to proxy the need for care [17–20, 22, 44]. Unfortunately, the variable on the days of illness in the past 2 weeks was hard to be applied to reflect children’s need for inpatient care in the last year. This omission might bias the estimates in the inpatient model, since children’s need for care was not only an important predictor for children’s use of care but also correlated with other explanatory variables. Though it would be nice to conclude whether the estimate on parental use of inpatient services had an upward or downward bias, the conclusion was not possible without further assumptions.

The limitation of using the days of illness in the past 2 weeks to measure children’s need for care was partially mitigated by adding into all regressions two other need variables reflecting overall child health: children’s height and weight [56, 57]. The results showed that children with low weight (conditional on height and age) had more inpatient visits last year and those with low height (conditional on weight and age) were more likely to use infusion during outpatient visits in the past 2 weeks. Consistent with the results of using the days of illness, these findings suggested that children with relatively poor health status used more health care services. We acknowledge that more accurate information on children’s need for care would improve the quality of the study. Nevertheless, with respect to the focused variable in our study, many previous studies have shown that parental use of health care was a significant predictor for children’s health care utilization after controlling for children’s need for care [17–19, 22].

The results showed that the number of children’s outpatient visits in the past 2 weeks and the probability of using infusion during outpatient visits were negatively associated with parental days of illness in the past 2 weeks. One hypothesis was that healthy parents were more capable of providing sufficient care to children including taking them to hospital and making medical decisions. Some previous studies which used yearly information on parental health status showed that healthy parents raised healthy children who used fewer health care services [17, 22]. However, as parental need for care was measured for the past 2 weeks in this study, we found no evidence for such a relationship, which might be more likely to exist in the long run.

Another interesting finding was that urban children used more outpatient care while rural children used more inpatient services. One speculation might be that urban children had a better access to high-quality outpatient care, which prevented the illness getting worse and therefore lowered the probability of hospitalization. Moreover, it has been found that inpatient visits per capita increased much faster than outpatient visits per capita in rural China after implementing NCMS which addressed little attention on outpatient care [14, 35].

We found that children’s use of health care services was negatively associated with their age. One explanation for this finding might be that younger children would be more vulnerable and therefore use more health care services. It might also be related to “fears” of new parents who tend to overuse health care services due to the lack of experience and knowledge [58, 59].

Parental age was shown to be positively related to the probability of using infusion during outpatient visits in the past 2 weeks and the number of inpatient visits last year. The association between parental age and children’s use of health care is theoretically unclear yet, but one hypothesis is that parental age may be positively correlated with the socioeconomic level of a family.

The results also showed that boys used more inpatient services than girls in our sample. Our results supported earlier findings that boys may use more health care services than girls [19, 20]. It is likely that boys report more injuries [14, 60]. In addition, previous studies showed that boys may have more biological health problems, such as respiratory diseases and autoimmune diseases, than girls [61, 62].

This study suffers from several limitations and raises several opportunities for future studies. First, in our study, only information on one parent, the head-of-household, was available. We were not able to conduct subsample analysis for the group of fathers and the group of mothers as most parents in our sample were fathers. With more information in the future, it would be interesting to see how the relationship between parental and children’s health care use may vary across these two groups. Second, given the current data, we were unable to assess how psychological factors were related to health care utilization as shown in previous studies [19, 23, 63]. To explore this issue, designing relevant questions should be specifically considered in future surveys. Third, our data did not allow us to test any hypothesis on the role of providers in generating supplier-induced overuse of health care services. More information on health provider characteristics, such as incentives, levels of training, and practice patterns, will be needed to study provider-induced moral hazard.

## Conclusions

With multivariate analyses, we found that children would use more outpatient care, infusion treatment, and inpatient care when their parents themselves used the corresponding medical services during the same time frame. The results suggested that Chinese children whose parents were high health care users were more likely to overuse health care services, holding other factors constant. Our findings imply that parents can play an important role in reducing children’s unnecessary health care utilization. The key is to change parental health-seeking beliefs and behaviors. Possible and effective communication strategies (such as the use of mass media and short message service text) should be explored to educate parents, especially parents of young children, on the potential adverse effects of overusing medications.

## Abbreviations

MEPS: Medical expenditure panel survey
NCMS: New rural Cooperative Medical Scheme
NHSS: National Health Service Survey
OLS: Ordinary least squares
URBMI: Urban Resident Basic Medical Insurance Scheme
